# Rapid Clinical Deterioration With Sepsis and Persistent Hypertension in a Pediatric Patient During Recent COVID-19 Crisis

**DOI:** 10.7759/cureus.14281

**Published:** 2021-04-03

**Authors:** Sabita Bhatta, Abida Sayed, Raj Kumar Bhatta, Yogesh Acharya

**Affiliations:** 1 Pediatrics, Woodhull Medical Center, New York, USA; 2 Medicine and Research, Avalon University School of Medicine, Willemstad, CUW; 3 Neurosurgery, Postgraduate Institute of Medical Education and Research, Chandigarh, IND; 4 Vascular and Endovascular Surgery, Western Vascular Institute, Galway, IRL

**Keywords:** 2019 novel coronavirus disease, covid-19, children, sepsis, hypertension

## Abstract

We present a child with a clear and classic COVID-19 symptomatic picture that rapidly progressed to sepsis with persistent hypertension. This patient, a five-year-old Hispanic female child was brought to our emergency department on March 21, 2020, with fever, productive cough, shortness of breath with chest tightness, abdominal pain, and diarrhea for a week. Her condition deteriorated rapidly, and she developed sepsis within 24 hours, needing intensive care unit admission and ventilator support. She tested negative for COVID-19 Biofire ® nucleic acid tests (BioFire Diagnostics, Salt Lake City, Utah 84108 USA); however, she was recently exposed to COVID-19 cases at her school. This case highlights the importance of a high index of COVID-19 suspicion in children in the endemic areas despite negative COVID-19 tests for keeping a watchful eye to prevent sudden deterioration and unexpected complications.

## Introduction

The coronavirus disease 2019 (COVID-19) pandemic has affected thousands of children globally in a short period, necessitating a critical understanding of the disease for its recognition [1]. Generally, children and young adults seem to be at lower risk; however, there are many recent cases where children had atypical presentations and unexpected complications [2, 3]. Children were thought to be less susceptible to COVID-19 at the beginning of the pandemic, but as new cases are unfolding rapidly, many cases of children, including neonates and teenagers, are progressively being reported in the literature [4]. Most children acquire the disease after direct contact with COVID-19 family members or close contacts [5]. Here, we report a pediatric case with a clinical picture consistent with COVID-19 that rapidly progressed to sepsis with persistent hypertension.

## Case presentation

A five-year-old Hispanic female child was brought to our emergency department on March 21, 2020, with fever (100.8 ℉), productive cough, shortness of breath, chest tightness, abdominal pain, and diarrhea for a week.

She became ill initially five days back with a dry cough, which soon became productive. Her maximum recorded fever at home was 101 ℉, after which her mother contacted their primary care physician. She was started on ibuprofen and amoxicillin, but failed to improve and continued having fever and further worsening of the cough. After three days, she developed chest pain, shortness of breath, abdominal pain, and loose stool. There were no significant past medical and surgical history or illness in the family. However, her school had recent cases of COVID-19. Her birth was uneventful, immunization status up-to-date, and she achieved all her age-appropriate developmental milestones.

On examination, the patient was alert but anxious and uncomfortable. She was breathing fast and shallow, respiratory rate (RR) 22 breaths/min, with 95% oxygen saturation in room air. Her temperature (temp) was 100.8 ℉, heart rate (HR)118 bpm with a regular rhythm, and elevated blood pressure (BP) 128/65 mm Hg. Her body mass index (BMI) was high (22 kg/m2, > 99th percentile). Her chest examination showed bilateral (b/l) decreased air entry and crackles on both lungs with a mild b/l subcostal retraction. She had epigastric tenderness, but her belly was soft with normal bowel sounds. Her neurological status was intact, and her skin was warm, with a capillary refill time < 2 seconds.

**Figure 1:**
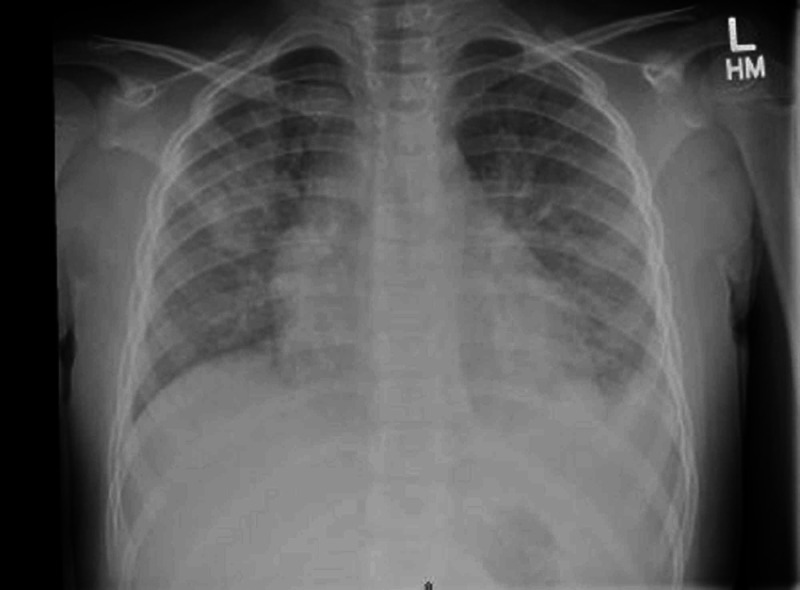
Chest x-ray on admission, showing a bilateral diffuse airspace opacities in the absence of multifocal pneumonia, pulmonary edema or acute respiratory distress syndrome

Radiological examination revealed b/l diffuse airspace opacities, likely multifocal pneumonia, pulmonary edema or acute respiratory distress syndrome (ARDS), and probable small left pleural effusion (Figure 1). Her initial full blood count, biochemical parameters, including renal function test (RFT) and liver function test (LFT), and arterial blood gas analysis (ABG) were within normal limits. However, her urine analysis showed gross hematuria and proteinuria (1000 mg/dl). She was admitted with a diagnosis of community-acquired pneumonia and ARDS, probably due to COVID-19 with acute kidney injury. She was started on antibiotics, azithromycin, and ceftriaxone (1500 mg twice daily). 

After admission, she worsened quickly over the next 24 hours with hypoxia (lowest ~ 88 %), tachycardia (HR:138 bpm), tachypnea (RR: 58 breaths/min), and elevated pressure (BP: 137/105 mm of hg). Respiratory support was initiated with high-flow nasal cannula (HFNC) (25 L/min Fio2 50 %) in a special care unit. Her work of breathing worsened in HFNC (RR: 65 and HR: 155, Spo2: 85-94%) and remained hypertensive (BP: 126/93). Anticipating severe respiratory failure, she was immediately transferred to the pediatric intensive care unit (PICU) on bi-level positive airway pressure (BiPAP). She progressively became lethargic with retracting and grunting, and she was immediately intubated. ARDS was managed with a ventilator setting peak end-expiratory pressure (PEEP) of 6 cm with a maximum PEEP of 10 cm, the fraction of inspiratory oxygen (FiO2) 50 %, low tidal volume 150 ml, and mean airway pressure of 12 cm.

Her rapid viral panel tests were positive for Rhino/Enterovirus. But her nasopharyngeal swab for COVID Biofire ® nucleic acid tests (BioFire Diagnostics, Salt Lake City, Utah 84108 USA) was reported negative. Her laboratory workup in intensive care unit (ICU) showed elevated white blood cell (WBC) 17.23x10^3/mcl (lymphocytes 13%, neutrophils 81%), C-reactive protein (CRP) 17.50 mg/dl (Normal <3 mg/dl ), procalcitonin 0.5 unit ng/ml, (normal range: 0.02-0.1 ng/ml), ferritin 321 ng/ml (normal 10-290 ng/ml), and d-dimer 571 units, (normal <230 ng/ml). Her coagulation profile was within the normal limits both at the admission and ICU. Arterial blood gas showed severe metabolic acidosis ABG [pH: 7.2, partial pressure of carbon dioxide (pCO2): 38, pO2:103, bicarbonate (HCO3):14, base excess: -11.6, lactate: 1.7].

She was managed for sepsis due to superimposed bacterial infection and acute respiratory failure/ARDS due to viral infection with high clinical suspicion for COVID 19. Antibiotics given were ceftriaxone (1500 mg twice daily) and vancomycin (15 mg/kg qid). Azithromycin and hydroxychloroquine were started for presumptive COVID infection on day four of PICU admission, initially, 6.5 mg/kg (max 400 mg oral) twice daily, and on day five the dosage was lowered to 3.5 mg/kg (max 200 mg) twice daily for four days. Cefepime (50 mg/kg Q8 hrs) was introduced for broad-spectrum coverage due to a breakthrough in her fever on day six of PICU.

After a week of management in ICU ,her WBC went down (WBC 10x10^3mcg/l, neutrophils 71%, lymphocytes 17%) .CRP remained elevated to 25 mg/dl. However, LFT was elevated showing mild transaminitis [alkaline phosphatase (ALP): 242U/l, aspartate aminotransferase (AST): 125U/L, ALT: 77U/l] and renal function progressively declined with a rise in creatinine from baseline 0.3 mg/dl to 0.8 mg/dl. She was persistently hypertensive with SBP ~150 mm Hg and required nicardipine infusion (1.5 mcg/kg/min) during PICU stay. Hemoglobin dropped from baseline 15 mg/dl to 8 mg/dl, however didn't require a blood transfusion. Her blood and urine cultures were negative.

**Figure 2:**
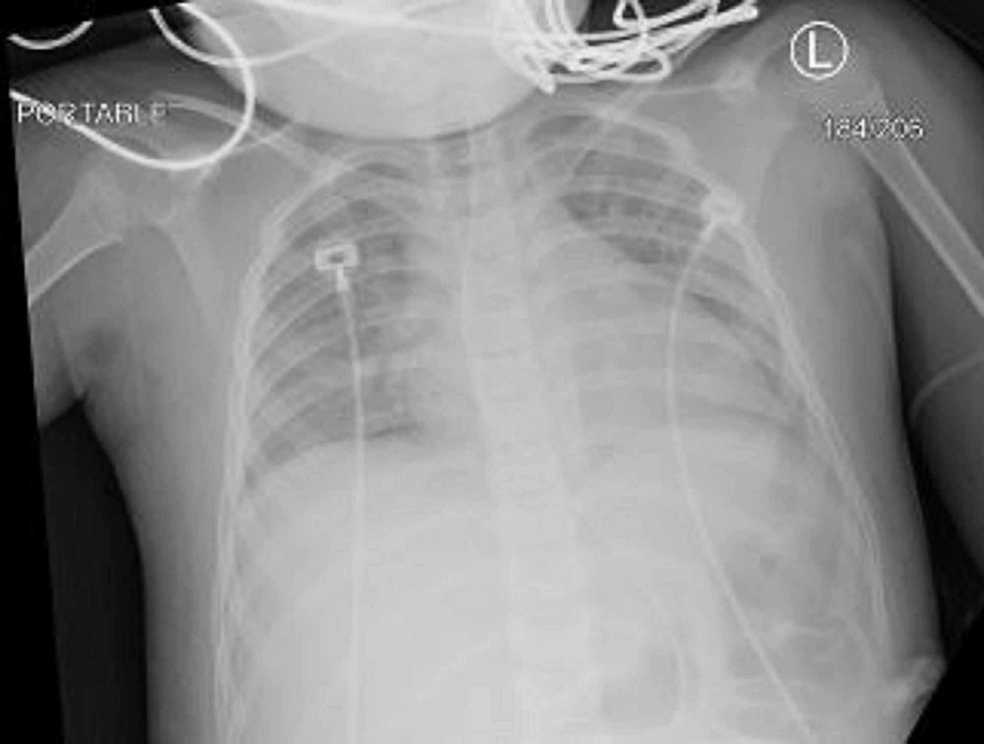
Chest x-ray post-extubation, showing signs of improved aeration with possible small airway inflammation as well as suspected small left effusion; however, assessment is limited by the low lung volumes.

**Figure 3:**
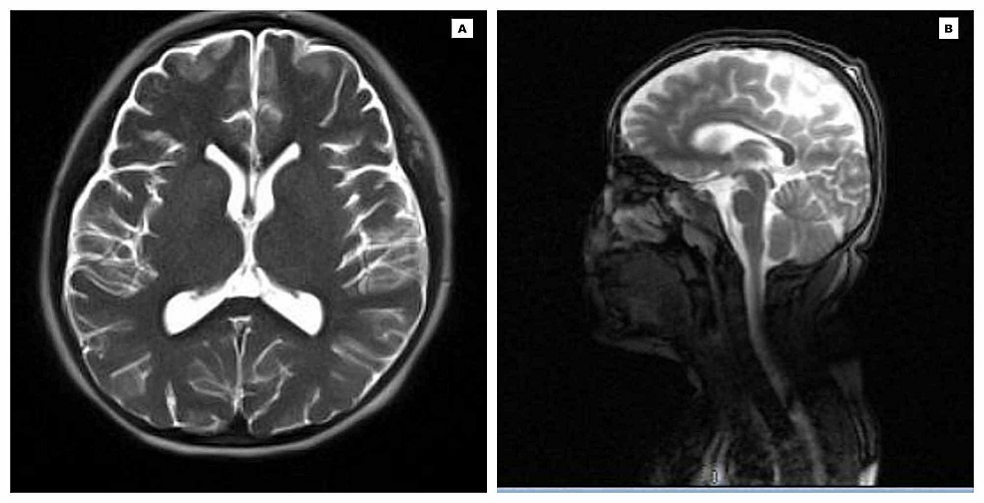
Magnetic Resonance Imaging of the head depicting normal findings with no acute intracranial abnormalities (A: Axial view; B: Sagittal view)

On April 2, 2020, her CXR (Figure 2) showed improvement in lung fields, CRP declined (15 mg/dl), and LFT (ALP: 146 U/l, AST: 30U/L, ALT:17U/L), RFT (creatinine: 0.4) and ABG normalized (pH: 7.4, pCO2: 30, pO2:83, HCO3:22, base excess: -1.3, lactate: 0.5). She progressively improved and was extubated on day 10 of the PICU. On post-extubation, she had decreased alertness, expressive aphasia, and cogwheel rigidity. Magnetic resonance imaging (MRI) (Figure 3), and video electroencephalogram (EEG) were done to rule out CNS pathology which was read normal.

Her residual symptoms resolved spontaneously however her blood pressure remained elevated. She was shifted to the floor on fifteen days of ICU stay following the completion of the two weeks of antibiotics course. She was continued with antihypertensive medications and discharged on April 10, 2020, with amlodipine [5 mg once a day (OD)], to be followed in a month for nephrology consultation.

## Discussion

The COVID-19 in children can present in varying forms, ranging from asymptomatic to mild-moderate illness with symptoms like fever, fatigue, dry cough, upper respiratory involvement with nasal obstruction and discharge, sore throat, as well as gastrointestinal involvement with vomiting, abdominal discomfort/pain, and diarrhea [6, 7]. In more severe cases, children presented with dyspnea, cyanosis, and signs of poor feeding. However, the majority of infected children recover within one to two weeks duration after symptom onset [6]. 

The COVID-19 illness is generally milder in children compared to adults; however, we can not ignore the possibility that it can rapidly progress to sepsis becoming fatal [5, 8, 9]. Early data in COVID-19 has shown severe respiratory manifestation in adults; however, limited data exist about pediatrics [10]. In the United States, approximately 0.58 - 2% of hospitalized pediatric patients were admitted to the ICU [11]. Dong et al. reported 6.7% and 0.7% severe and critical cases in children, respectively. Severe diseases are usually seen in infants and children with underlying severe systemic or immunological illnesses [12]. The case fatality rate is less than 1%, and the prevalence of extreme cases, approximately less than 10% among infected children [5].

Our patient rapidly progressed to sepsis, requiring ventilatory support. She also had acute kidney injury with increased blood pressure throughout the course. As the SARS-CoV 2 uses ACE2 receptors in the kidney for cellular entry and attachment, there is a possibility of upregulation of the renin-angiotensin-aldosterone system (RAAS) with renal impairment and hypertension. However, we believe there are many frontiers of these intricate complexities between the tri-junction of RAAS, renal impairment, and hypertension that are yet to unfold. For now, we did not find reports of persistent hypertension in COVID-19 pediatric patients.

Diagnosis rests on the clinical picture and evidence of infection through a pharyngeal swab that detects COVID-19 viral nucleic acid [13]. Unfortunately, the positive rate is proportionately lower, particularly during the early phases of the illness due to the limitations in sampling material. Many patients showing a negative nucleic acid test were treated based on radiological evidence. In our case, Biofire test® (BioFire Diagnostics, Salt Lake City, Utah 84108 USA) taken from the nasopharyngeal swab, for SARS-CoV2 viral RNA for COVID-19 (sensitivity 97.1% and specificity 99.3%) was negative. However, as our patient had clinical signs and symptoms consistent with COVID-19 and history of exposure to suspected cases in endemic areas, we admitted her with a presumptive COVID-19 diagnosis. Her rapid viral panel was positive for ‘Rhino’ and ‘Enterovirus.’ Previous studies have revealed that children can have viral co-infections in COVID-19 up to two-thirds of cases [14] has severe disease course.

Similarly, COVID-19 can have variable laboratory findings in children. Most of the reported pediatric patients have shown normal WBC and lymphocyte counts. This was also observed in a retrospective observational study by Xia et al. involving 20 pediatric COVID-19 patients [13]. They found that the majority of their patients (70%) showed normal WBC findings. Another retrospective case series of 67 patients involving both adults and children showed that during the early phases of COVID-19, there was no significant decline in lymphocyte count; however, neutrophils decreased in children compared to adults (P=0.02) [15]. In our case, the patient had normal WBC and lymphocytes on the day of admission, but her overall WBCs and neutrophils became elevated as a result of sepsis on the second day.

Previous reports indicated that decreased lymphocyte counts were common, especially in severe cases [7, 8]. As a result of viral infection, white blood cell and lymphocyte counts can be reduced by consumption. However, similar results did not appear in our study, with only a total of six cases, including one case of a child with decreased lymphocyte counts. The value of lymphocyte counts showed no significant differences in children over six years compared with adults. On the other hand, neutrophil counts decreased in children compared with adults (P=0.02). So, lymphocyte decline is not an essential indicator for the diagnosis of childhood cases, and the neutrophil count decline should be focused on. These may be due to a lower inflammatory response in children. 

Management of children with worsening symptoms includes airway and oxygen therapy with the use of nasal cannula in young children [16]. The standard management protocol of COVID-19 lacks in children. The Zhejiang University School of Medicine has recommended intravenous immunoglobulin in severe cases and nebulized interferon alpha-2b, oral lopinavir/ritonavir, and corticosteroid for severe complications like ARDS, encephalitis, hemophagocytic syndrome, or septic shock [11]. However, there is no clear clinical evidence to support these therapies. Therefore, both the World Health Organization (WHO) and the Centers for Disease Control and Prevention (CDC) have no definitive treatment recommendations following COVID-19 in children [17, 18].

It is important to monitor patients, especially children with COVID-19 infection, for sudden deterioration, rapid and progressive respiratory distress, and sepsis with immediate follow-up with supportive care. In our case, the child did not test positive for COVID-19, but the symptoms were consistent with it, and her general condition rapidly deteriorated, needing PICU admission and intubation. Many of these clinical worsenings may be attributed to co-infection with other viruses. This study highlights the possibility of rapid deterioration and sepsis in a child in a suspected COVID-19 case despite negative viral evidence. Therefore, it is crucial to consider the presumptive diagnosis of COVID-19 in every child with a typical clinical picture and suspected history of exposure, particularly from current endemic areas, and remain vigilant in its progression. 

## Conclusions

Children with COVID-19 can present with variable signs and symptoms. Our case shows an example of a clinically symptomatic COVID-19 picture without viral evidence that rapidly deteriorated to develop sepsis with significant kidney injury and persistent hypertension. It is crucial to have a watchful eye on every child with suspicion of COVID-19, especially in high endemic areas, even if the COVID-19 testing is negative. Clinical pictures, radiological imaging, and supportive inflammatory markers are needed to evaluate the patient. High clinical suspicions and vigilance is required to prevent rapid clinical deterioration and unexpected complications.
